# BCA2/Rabring7 Interferes with HIV-1 Proviral Transcription by Enhancing the SUMOylation of IκBα

**DOI:** 10.1128/JVI.02098-16

**Published:** 2017-03-29

**Authors:** Marta Colomer-Lluch, Ruth Serra-Moreno

**Affiliations:** Department of Biological Sciences, College of Arts and Sciences, Texas Tech University, Lubbock, Texas, USA; Emory University

**Keywords:** NF-κB, virology, host-pathogen interactions, human immunodeficiency virus, innate immunity

## Abstract

BCA2/Rabring7 is a BST2 cofactor that promotes the lysosomal degradation of trapped HIV-1 virions but also functions as a BST2-independent anti-HIV factor by targeting Gag for lysosomal degradation. Since many antiviral factors regulate the NF-κB innate signaling pathway, we investigated whether BCA2 is also connected to this proinflammatory cascade. Here, we show for the first time that *BCA2* is induced by NF-κB-activating proinflammatory cytokines and that upregulation of BCA2 provides regulatory negative feedback on NF-κB. Specifically, BCA2 serves as an E3 SUMO ligase in the SUMOylation of IκBα, which in turn enhances the sequestration of NF-κB components in the cytoplasm. Since HIV-1 utilizes NF-κB to promote proviral transcription, the BCA2-mediated inhibition of NF-κB significantly decreases the transcriptional activity of HIV-1 (up to 4.4-fold in CD4^+^ T cells). Therefore, our findings indicate that BCA2 poses an additional barrier to HIV-1 infection: not only does BCA2 prevent assembly and release of nascent virions, it also significantly restricts HIV-1 transcription by inhibiting the NF-κB pathway.

**IMPORTANCE** Understanding the interactions between HIV-1 and its host cells is highly relevant to the design of new drugs aimed at eliminating HIV-1 from infected individuals. We have previously shown that BCA2, a cofactor of BST2 in the restriction of HIV-1, also prevents virion assembly in a BST2-independent manner. In this study, we found that BCA2 negatively regulates the NF-κB pathway—a signaling cascade necessary for HIV-1 replication and infectivity—which in turn detrimentally affects proviral transcription and virus propagation. Thus, our results indicate that, besides its previously described functions as an antiviral factor, BCA2 poses an additional barrier to HIV-1 replication at the transcriptional level.

## INTRODUCTION

Restriction factors are cellular proteins that display autonomous and dominant effects to block the infection of virus pathogens (1). In the case of human immunodeficiency virus (HIV), six potent antiviral factors are currently known: TRIM5α/TRIMCyp (2–6), members of the APOBEC3 family (7–9), SAMHD-1 (10–12), MX2 (13–15), Tetherin/BST2 (16, 17), and the SERINC3 and SERINC5 proteins (18, 19). A recent study identified BCA2 (breast cancer-associated gene 2, also known as Rabring7, RNF115, or ZNF364) as a cofactor of BST2 in the restriction imposed on HIV-1 (20). However, we recently demonstrated that, besides its BST2-dependent activity, BCA2 also has BST2-independent anti-HIV-1 activity. Specifically, BCA2 promotes the ubiquitination and lysosomal degradation of retroviral Gag proteins, which impairs virus assembly (21).

Certain HIV-1 antiviral factors are connected to the NF-κB innate signaling pathway either by functioning as viral sensors (i.e., TRIM5α and Tetherin) or by responding to NF-κB (i.e., APOBEC3G) (22–27). Notably, the promoter region of *BCA2* also contains responsive elements for NF-κB (28), suggesting that *BCA2* is induced by NF-κB-activating signals. In addition to its role in the activation of proinflammatory responses (29–32), NF-κB is critical for the replication of HIV-1. HIV-1 contains NF-κB-responsive elements in the transcriptional control regions of its long terminal repeats (LTRs) (33, 34), and thus, NF-κB activation enhances the transcriptional activity of HIV-1 (35–37). Strikingly, HIV-1 ensures the induction of the NF-κB pathway by two different mechanisms: through gp41 (38) and Nef (39). Hence, HIV-1 takes advantage of this innate cascade to increase its propagation.

In this study, we demonstrate that *BCA2* is induced by NF-κB-activating proinflammatory cytokines and that upregulation of BCA2 provides regulatory negative feedback on this pathway. In particular, BCA2 prevents the nuclear translocation of NF-κB by increasing the SUMOylation of IκBα, an inhibitor of NF-κB. BCA2 outcompetes HIV-1 gp41 in the modulation of this cascade and reduces HIV-1 transcription by 4.4- and 2-fold in CD4^+^ T cell lines and primary cells, respectively, causing up to a 4-fold defect in virus replication. Taken together, these results indicate that, besides its BST2-dependent and -independent functions as an HIV-1 inhibitor, BCA2 poses an additional barrier to HIV-1 replication by inactivating the NF-κB pathway.

## RESULTS

### *BCA2* is induced by NF-κB-activating proinflammatory cytokines and provides regulatory feedback on NF-κB.

**FIG 1:**
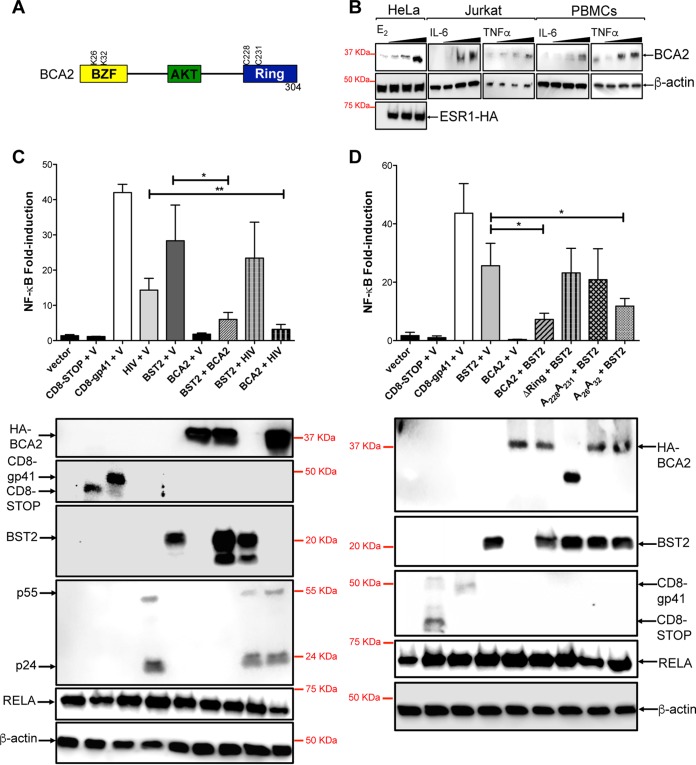
BCA2 is induced by NF-κB-activating cytokines, and its upregulation serves as regulatory feedback for NF-κB signaling. (A) Schematic representation of BCA2. Domains and important residues are indicated. (B) Jurkat CD4^+^ T cells and human PBMCs (10^6^) were treated with increasing concentrations of IL-6 (0.1 to 2 ng/ml) and TNF-α (0.1 to 10 ng/ml), and the expression levels of endogenous BCA2 relative to β-actin were determined by Western blotting 24 h later. As controls, vector-transduced parental HeLa cells were included, as well as HeLa cells stably expressing estrogen receptor (ESR1), and treated with estrogen (E_2_) (1 to 100 ng/ml). (C and D) 293T cells were cotransfected with an NF-κB reporter vector, a β-galactosidase reporter vector, and either a control pcDNA5 plasmid (V) or the indicated expression plasmids alone or in combination (pcDNA3-BST2, pcDNA5-HA-BCA2, pcDNA5-HA-A_26_A_32_, pcDNA5-HA-A_228_A_231_, pcDNA5-HA-ΔRing, pNL4.3, CD8-STOP, or CD8-gp41). Forty-eight hours later, luciferase activity was measured and normalized to β-galactosidase activity. (Bottom) The expression of each construct was confirmed by Western blotting. The data correspond to the mean and standard deviation of three biological replicates, measured in technical replicates. Values that are significantly different are indicated by asterisks (*, *P* ≤ 0.05; **, *P* ≤ 0.01). BZF, BCA2 zinc finger domain; AKT, AKT phosphorylation sites.

BCA2 was initially identified as a marker that positively correlates with invasive breast cancer and that is regulated by estrogen (28, 40, 41). This highly conserved protein is a RING finger E3 ubiquitin (Ub) ligase with two distinct domains: an N-terminal zinc finger domain (BZF), which binds ubiquitin and is susceptible to becoming ubiquitinated, and a C-terminal RING finger domain that catalyzes ubiquitination of BCA2-interacting partners and/or autoubiquitination. In addition, BCA2 harbors two AKT phosphorylation sites (Fig. 1A) (42). Besides being regulated by estrogen (28, 41), *BCA2* may also be controlled by NF-κB, since there are NF-κB-responsive elements in its promoter (28). In order to test this, the expression of *BCA2* was examined in Jurkat CD4^+^ T cells, as well as in human peripheral blood mononuclear cells (PBMCs), in response to different NF-κB-activating proinflammatory cytokines, such as interleukin 6 (IL-6) and tumor necrosis factor alpha (TNF-α) (43, 44). HeLa cells transduced with an empty vector or estrogen receptor (ESR1) and treated with estrogen (estradiol or E_2_) were used as a positive control. Remarkably, all these treatments led to the upregulation of BCA2 (Fig. 1B), confirming that *BCA2* is induced by NF-κB-activating proinflammatory signals.

To study the effect of BCA2 on the NF-κB signaling pathway, a luciferase-based assay was utilized (38). 293T cells were cotransfected with a construct coding for hemagglutinin-tagged BCA2 (HA-BCA2) along with a luciferase vector with NF-κB-responsive elements in the promoter and a β-galactosidase vector, the expression of which was used for protein normalization (38). As negative controls, constructs that did not affect NF-κB were used (an empty vector and a deleted version of CD8 [38]). As positive controls, reported inducers of NF-κB activity were included, such as a construct coding for the cytoplasmic domain of HIV-1 gp41 (CD8-gp41) (38), HIV-1 NL4.3 proviral DNA (33), and BST2 (22, 25). As expected, the presence of CD8-gp41, HIV-1, and BST2 strongly activated NF-κB, whereas expression of HA-BCA2 resulted in NF-κB activity levels similar to those observed with the negative controls (Fig. 1C). To elucidate if BCA2 negatively regulates NF-κB or if it does not affect this pathway, cells were cotransfected with constructs of NF-κB inducers (BST2 and HIV-1 proviral DNA) and HA-BCA2, and the status of the NF-κB pathway was analyzed by luciferase expression. A reduction of ∼80% in NF-κB activity was observed in these two scenarios (Fig. 1C), indicating that BCA2 inhibits NF-κB responses. Next, we evaluated whether the BCA2-mediated inactivation of this pathway requires the catalytic activity of BCA2. To test this, additional luciferase assays were performed in 293T cells coexpressing BST2, since BST2 is a robust NF-κB inducer, and HA-BCA2 mutants with impaired E3 ligase function, such as a ΔRing mutant (in which the entire RING finger domain was deleted [20, 21]) and a mutant containing amino acid substitutions in the E3 catalytic domain (C_228_ and C_231_ to A_228_ and A_231_). A zinc finger mutant (K_26_ and K_32_ to A_26_ and A_32_) was included as a control (21, 42) (Fig. 1A). Notably, NF-κB activity was rescued in the presence of the RING finger mutants (Fig. 1D). Hence, BCA2 requires a functional RING finger domain to turn off the NF-κB pathway.

### BCA2 modulates other innate immune transcription factors.

**FIG 2:**
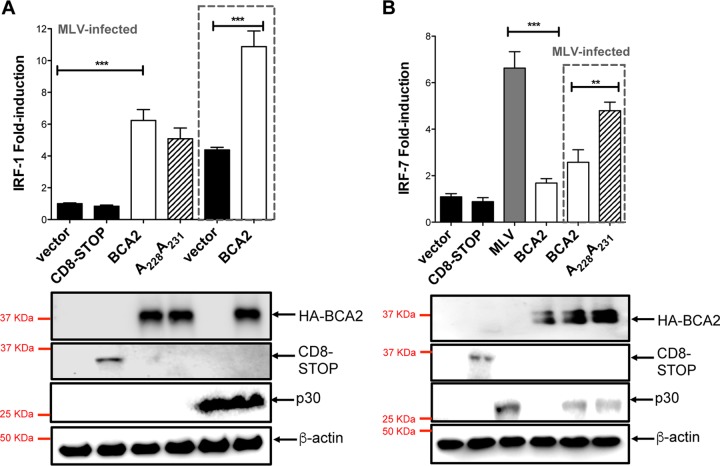
BCA2 regulates IRF-1- and IRF-7-mediated responses. 293T cells were cotransfected with an IRF-1 reporter vector (A) or an IRF-7 reporter plasmid (B), along with a β-galactosidase reporter vector and either an empty vector (pcDNA5), pcDNA5-HA-BCA2, or pcDNA5-HA-A_228_A_231_. Similar assays were performed in the presence of VSV-G-pseudotyped Mo-MLV. Forty-eight hours posttransfection, luciferase activity was measured and normalized to β-galactosidase activity. (Bottom) The expression of each construct was confirmed by Western blotting. The data correspond to the mean and standard deviation of three biological replicates, measured in technical replicates. The dashed lines indicate assays under conditions of MLV infection. Values that are significantly different are indicated by asterisks (**, *P* ≤ 0.01; ***, *P* ≤ 0.001).

To assess if BCA2 could similarly affect the functionality of other transcription factors involved with innate immunity, we investigated the effect of BCA2 on interferon regulatory factors, such as IRF-1 and IRF-7. Whereas IRF-7 is activated in response to viral infections and NF-κB (45), IRF-1 responses are mainly triggered by IFN-γ, although they can also be activated by cytosolic pattern recognition receptors (46, 47). To examine the implication, if any, of BCA2 in the regulation of IRF-1 and IRF-7, we conducted additional luciferase reporter assays. For this, 293T cells were cotransfected with IRF-1 or IRF-7 luciferase reporter vectors, the β-galactosidase plasmid, and a construct coding for HA-BCA2 or a BCA2 RING-defective mutant (A_228_ A_231_). An empty pcDNA5 vector and the CD8-STOP construct were included as negative controls. The assays were performed in the presence and absence of murine leukemia virus (MLV) infection as a control for the activation of both IRF-1 and IRF-7 responses. Expression of HA-BCA2 led to a significant increase in IRF-1 signaling, and unlike its effect on NF-κB, activation of IRF-1 was independent of BCA2's catalytic activity, suggesting that the regulatory elements for the modulation of NF-κB and IRF-1 are genetically separable (Fig. 2A). Remarkably, the BCA2-mediated induction of IRF-1 responses was even more pronounced in the context of MLV infection. By contrast, overexpression of BCA2 significantly hindered IRF-7-mediated activation, even in the presence of retroviral infection, a known inducer of this pathway (Fig. 2B). Similar to its effect on NF-κB, BCA2 requires a functional RING finger domain in order to effectively inhibit IRF-7 (Fig. 2B). Hence, besides its implication in the regulation of NF-κB, BCA2 plays additional roles in the modulation of innate signaling cascades.

### BCA2 prevents HIV-1-dependent activation of the NF-κB pathway, negatively affecting HIV-1 transcription.

**FIG 3:**
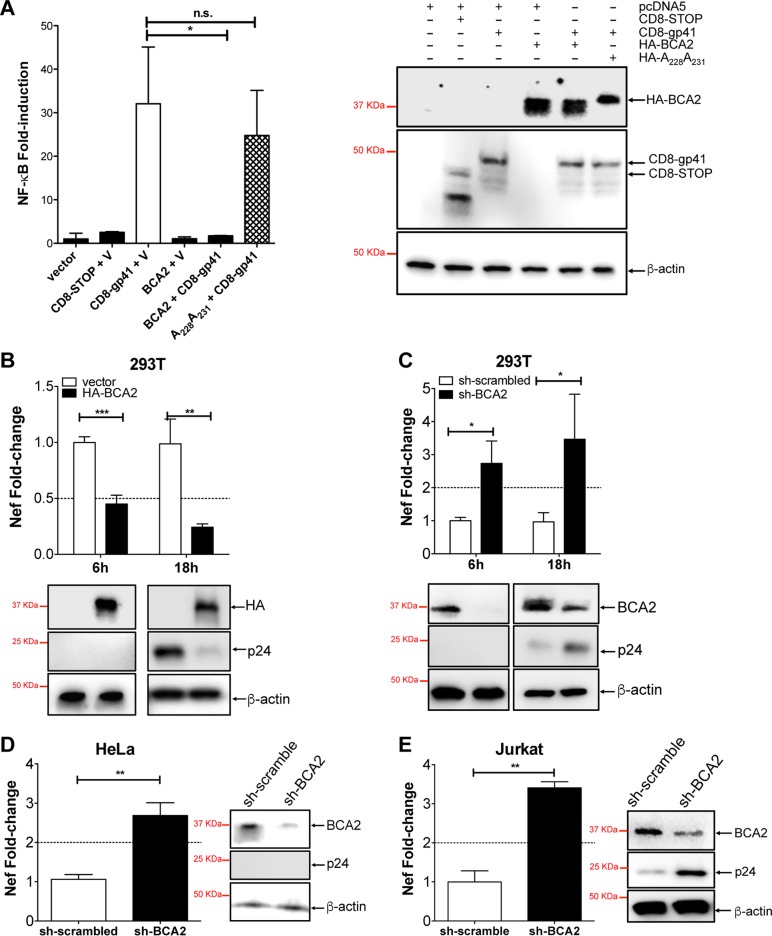
BCA2 prevents HIV-1-induced activation of the NF-κB pathway and restricts HIV-1 transcription. (A) Luciferase assays were conducted in 293T cells transfected with an empty pcDNA5 vector (V) or constructs coding for CD8-STOP, HA-BCA2, or a BCA2 RING-defective mutant (A_228_A_231_) in the presence or absence of CD8-gp41. (Right) Levels of expression of the indicated constructs. (B) The relative transcription of HIV-1 was determined in 293T cells overexpressing HA-BCA2 transfected with HIV-1 NL4.3 proviral DNA. For this, the amount of *nef*-harboring transcripts was assessed by RT-qPCR 6 and 18 h after proviral treatment. (C) BCA2-depleted 293T cells were transfected with HIV-1 NL4.3 proviral DNA, and the levels of *nef* RNAs were analyzed 6 and 18 h later by RT-qPCR. (D) Similar assays were performed in BCA2-depleted HeLa cells 6 h after HIV-1 NL4.3 proviral transfection. (E) Depletion of endogenous BCA2 was also achieved in Jurkat CD4^+^ T cells by lentiviral transduction. The cells were then infected with HIV-1 NL4.3, and the amount of *nef*-containing transcripts was determined by RT-qPCR 18 h later. The cell lysates derived from these experiments were analyzed by Western blotting for β-actin, BCA2, and p24 to evaluate the presence or absence of capsid at the time of analysis. The data correspond to the mean and standard deviation of three biological replicates, measured in technical replicates. Values that are significantly different are indicated by asterisks (*, *P* ≤ 0.05; **, *P* ≤ 0.01; ***, *P* ≤ 0.001; n.s., not significant).

It has long been known that HIV-1 and other primate lentiviruses induce the NF-κB cascade to facilitate proviral transcription, since they harbor NF-κB-responsive elements in the regulatory regions of their LTRs (33, 34). Our data indicate that upregulation of BCA2 hinders NF-κB activity, even in the presence of HIV-1 provirus. Therefore, we reasoned that the BCA2-mediated suppression of NF-κB has detrimental effects on HIV-1 replication. To test this hypothesis, HA-BCA2 was coexpressed in 293T cells along with a construct containing the cytoplasmic domain of HIV-1 gp41 (CD8-gp41), known to induce the canonical pathway of NF-κB (38). Whereas CD8-gp41 led to more than 30-fold induction in NF-κB activity in the absence of HA-BCA2, coexpression of CD8-gp41 and HA-BCA2 resulted in the complete inhibition of this cascade, indicating that BCA2 prevents HIV-1-dependent activation of NF-κB (Fig. 3A). By contrast, coexpression of the BCA2 RING-defective mutant and CD8-gp41 had no significant impact on the ability of gp41 to induce this pathway, supporting the need for BCA2's catalytic activity to block NF-κB responses (Fig. 3A).

To assess whether BCA2 upregulation has detrimental effects on the transcriptional capacity of HIV-1, the amount of *nef*-containing transcripts was measured by reverse transcription (RT)-quantitative PCR (qPCR). qPCR primers specific for HIV-1 *nef* were designed to compare proviral RNA levels in vector-treated and HA-BCA2-expressing 293T cells transfected with HIV-1 NL4.3 proviral DNA. Remarkably, cells overexpressing HA-BCA2 showed a 2.3-fold reduction in HIV-1 transcription only 6 h after proviral transfection and over 4-fold reduction in *nef*-containing transcripts 18 h later (Fig. 3B). To corroborate these observations, similar experiments were performed under conditions of BCA2 depletion. Briefly, 293T cells were transfected with lentivirus-based vectors coding for BCA2-specific short hairpin RNAs (shRNAs) or scrambled shRNA. The knockdown of BCA2 was verified by Western blotting using a BCA2-specific antibody and comparing the expression of endogenous BCA2 to that of β-actin. Next, cells were transfected with HIV-1 NL4.3 proviral DNA. The transcriptional activity of HIV-1 was analyzed by measuring the amount of *nef*-harboring RNAs 6 and 18 h later. In support of the data presented above, the targeted depletion of BCA2 resulted in a 2.7- and a 3.4-fold increase in viral transcription, respectively (Fig. 3C). Similar results were obtained in BCA2 knockdown HeLa cells 6 h after proviral transfection, with an average of 2.68-fold induction in viral transcription (Fig. 3D), and in Jurkat CD4^+^ T cells depleted of BCA2. In this case, cells transduced with scrambled shRNA or BCA2-specific shRNAs were infected with 100 ng of p24 equivalents of HIV-1 NL4.3, and the levels of *nef* transcripts were evaluated by RT-qPCR 18 h postinfection. Consistent with our previous results, the amounts of viral RNAs increased by 3.4-fold in BCA2 knockdown Jurkat CD4^+^ T cells (Fig. 3E). Thus, BCA2 negatively affects HIV-1 proviral transcription.

**FIG 4:**
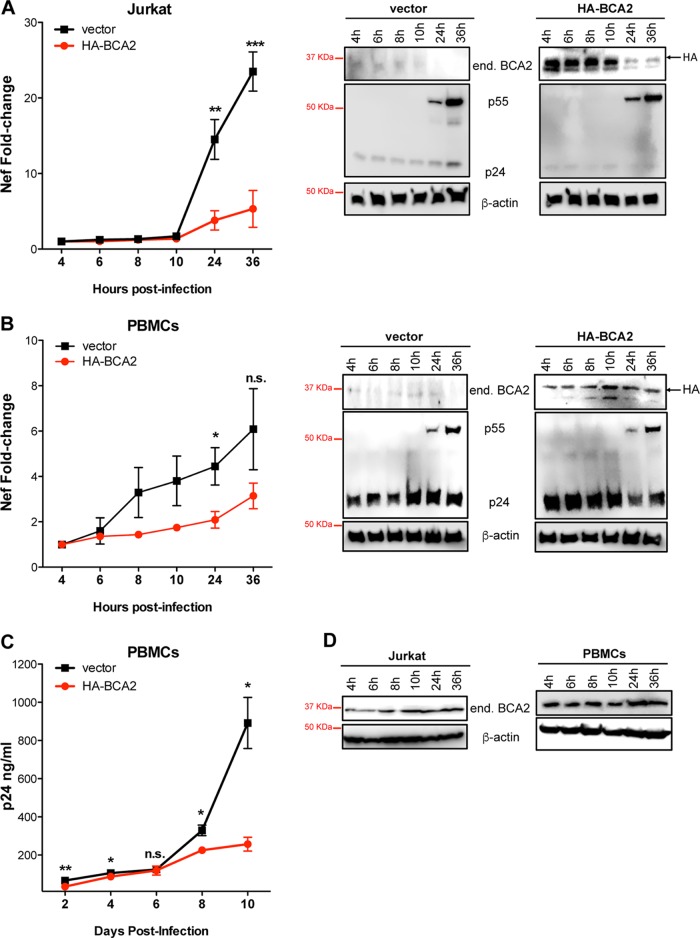
The BCA2-mediated block on NF-κB delays proviral transcription and replication in CD4^+^ T cells. Jurkat CD4^+^ T cells (A) and ConA-activated and IL-2-expanded human PBMCs (B) were transduced with a vector encoding HA-BCA2 or treated with an empty vector (pQCXIP). Forty-eight hours later, the cells were infected with HIV-1 NL4.3. Proviral transcription was assessed at the selected time points by measuring *nef* RNA levels by RT-qPCR. (Right) The cell lysates derived from these experiments were analyzed by Western blotting for β-actin, BCA2, and p55/p24. (C) HIV-1 NL4.3 replication was also assessed in PBMCs transduced with an empty vector or HA-BCA2 by measuring the amounts of capsid p24 released to the culture supernatant at days 2, 4, 6, 8, and 10 postinfection. (D) The endogenous levels of BCA2 and β-actin were monitored over time in uninfected Jurkat CD4^+^ T cells and human PBMCs. The data correspond to the mean and standard deviation of three biological replicates, measured in technical replicates. Values that are significantly different are indicated by asterisks (*, *P* ≤ 0.05; **, *P* ≤ 0.01; ***, *P* ≤ 0.001; n.s., not significant).

To further investigate the relevance of BCA2 to the transcriptional activity of HIV-1, additional replication assays were conducted in Jurkat CD4^+^ T cells and human PBMCs transduced with either a retroviral empty vector (pQCXIP) or a retroviral construct coding for HA-BCA2. Two days later, the cells were infected with 100 ng of p24 equivalents of HIV-1 NL4.3, and proviral transcription was evaluated by assessing the amounts of *nef*-containing transcripts by RT-qPCR at 4, 6, 8, 10, 24, and 36 h postinfection. In addition, virus replication was assessed in PBMCs by measuring the amounts of capsid p24 released to the culture supernatant. In agreement with our previous results, overexpression of BCA2 caused a major delay in proviral transcription in both cell types, particularly at 24 and 36 h postinfection, and also in virus replication (∼3.5-fold reduction) (Fig. 4A to C), reflecting the fact that BCA2's regulatory effect on NF-κB poses a hurdle to HIV-1 transcription. Nevertheless, a reduction in the overall protein levels of both endogenous BCA2 and HA-BCA2 was detected 24 and 36 h postinfection (Fig. 4A and B, right). To assess if the decline in BCA2 is a result of its own half-life or a consequence of viral infection, uninfected Jurkat CD4^+^ T cells and human PBMCs were seeded at the same concentrations as for the infectivity assays, and the endogenous levels of BCA2 were monitored at the selected time points by Western blotting. Compared to β-actin, no significant differences in the protein levels of BCA2 were detected over time in uninfected cells (Fig. 4D), suggesting that the observed reduction in BCA2 is a consequence of viral infection. This effect was more pronounced in Jurkat CD4^+^ T cells than in PBMCs, probably due to the presence of a mixed population of white blood cells in the latter, which reflects the fact that not all PBMCs are susceptible to HIV-1 infection, and thus, less degradation of BCA2 is observed. Therefore, our results indicate that either HIV-1 has evolved to partially circumvent the block imposed by BCA2 (by decreasing BCA2's steady-state levels) or the observed reduction in BCA2 is a side effect of the viral infection, probably due to the activation of additional innate immune pathways that may cause a decrease in BCA2.

### BCA2 requires UBC9, but not UbcH5, to inhibit the NF-κB pathway and limit HIV-1 transcription.

**FIG 5:**
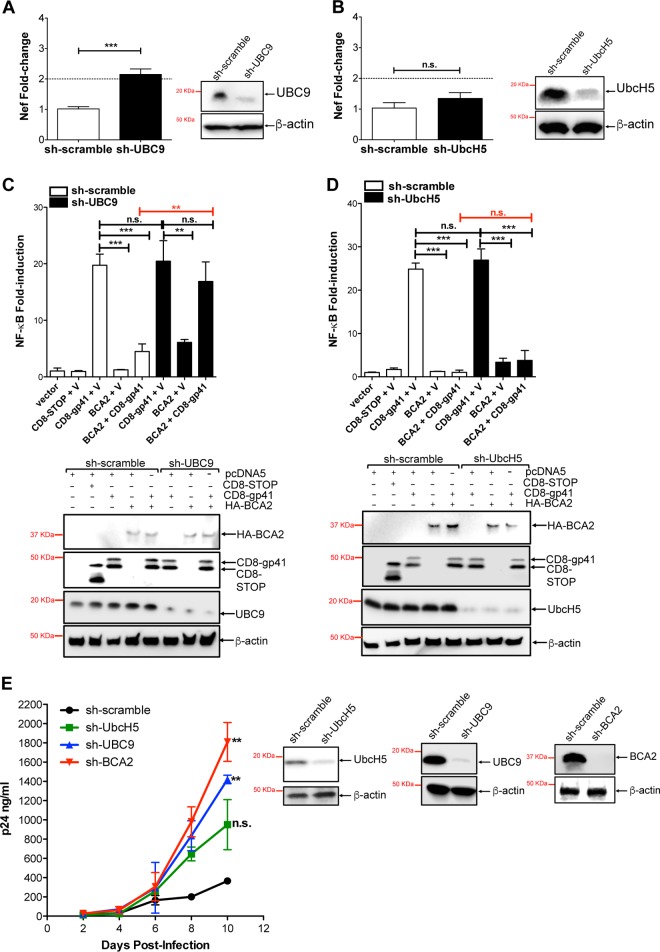
BCA2 requires UBC9, but not UbcH5, to inhibit NF-κB and restrict HIV-1 transcription. (A) UBC9-depleted 293T cells were transfected with HIV-1 NL4.3. Total RNA was isolated 6 h posttransfection, and the presence of *nef*-containing RNAs was assessed by RT-qPCR. (B) Similarly, *nef* RNA levels were assessed in UbcH5-depleted 293T cells. (C and D) Luciferase assays were performed in 293T cells that were depleted of UBC9 (C) or UbcH5 (D) in the presence of HA-BCA2 and CD8-gp41. NF-κB activity is expressed as fold induction after β-galactosidase normalization. (Bottom) The expression of β-actin, HA-BCA2, UBC9, UbcH5, and CD8-based constructs was verified by Western blotting. (E) HIV-1 replication was monitored by p24 antigen capture ELISA in Jurkat CD4^+^ T cells specifically depleted of BCA2, UBC9, or UbcH5. Western blot analyses corroborated depletion of these proteins at day zero of infection. The data correspond to the mean and standard deviation of three biological replicates, measured in technical replicates. Values that are significantly different are indicated by asterisks (**, *P* ≤ 0.01; ***, *P* ≤ 0.001; n.s., not significant). V, vector.

Since the results presented in Fig. 1D indicate that BCA2 requires a functional RING finger domain in order to effectively reduce NF-κB activity, additional transcriptional assays were performed in 293T cells under conditions of knockdown of UBC9 or UbcH5, the two known E2-conjugating enzymes that interact with BCA2 (28, 48). HIV-1 transcription increased 2.2-fold in UBC9 knockdown cells 6 h after HIV-1 proviral transfection (Fig. 5A). However, in UbcH5-depleted cells there was a modest 1.34-fold increase in the amount of *nef*-containing transcripts (Fig. 5B). To confirm that UBC9 is the E2-conjugating enzyme required for BCA2-mediated inhibition of NF-κB, additional NF-κB-based luciferase assays were conducted in the presence of shRNAs that targeted these BCA2-interacting E2 enzymes. Under conditions of HA-BCA2 overexpression and UBC9 knockdown, a slight increase in NF-κB activity was observed compared to sh-scrambled-treated cells (Fig. 5C, 4th bar from left versus 7th bar). Remarkably, in UBC9-depleted cells coexpressing HA-BCA2 along with CD8-gp41, NF-κB activity was restored: luciferase induction levels were similar to those observed in cells coexpressing CD8-gp41 and sh-scrambled or sh-UBC9 RNAs (Fig. 5C, 8th bar versus 3rd and 6th bars). By contrast, no defects in the ability of BCA2 to inhibit NF-κB were observed under conditions of UbcH5 depletion (Fig. 5D). Therefore, these results reveal that BCA2 uses UBC9 as the E2-conjugating enzyme to hinder NF-κB and limit HIV-1 transcription.

We previously reported that BCA2 promotes the ubiquitination and subsequent lysosomal degradation of HIV-1 Gag in a BST2-independent manner (21). In order to achieve this, BCA2 uses UbcH5 as the E2 Ub-conjugating enzyme. However, our data indicate that UbcH5 is dispensable for BCA2-mediated inhibition of NF-κB (Fig. 5D). To investigate what the contribution of each activity of BCA2 is to HIV-1 restriction (Gag degradation versus NF-κB inhibition), replication assays were performed in Jurkat CD4^+^ T cells depleted of BCA2, UBC9, or UbcH5. Depletion of UbcH5 resulted in a 2.7-fold increase in virus replication by day 10 postinfection. Remarkably, the selective knockdown of UBC9 afforded a 4-fold increase in virus replication. Consistent with our previous studies, BCA2 depletion led to a 5-fold increase in p24 levels at day 10 postinfection (21). Hence, according to our results, the inhibition of NF-κB would account for most of the antiviral activity of BCA2 (Fig. 5E).

### BCA2 enhances the SUMOylation of IκBα to block NF-κB responses.

As indicated above, BCA2 physically interacts with UBC9, an E2-conjugating enzyme for SUMO (small ubiquitin-like modifier) (28). Besides serving as a conjugating enzyme, UBC9 can also mediate ligation of SUMO in the absence of a matching E3 ligase, although the efficiency rate of this reaction significantly increases in the presence of an E3 enzyme (49, 50). UBC9 plays an important role in the regulation of NF-κB by promoting the SUMOylation of IκBα, an inhibitor of the NF-κB canonical pathway that prevents the translocation of NF-κB to the nucleus. In most nonstimulated cells, IκBα interacts with NF-κB molecules, hiding their nuclear localization signal (NLS), which in turn leads to their retention in the cytoplasm (51–53). However, under certain stimuli, IκBα becomes phosphorylated, and this posttranslational modification facilitates its ubiquitination, leading to its proteasomal degradation (54, 55). Therefore, the breakdown of IκBα allows the nuclear translocation of NF-κB. In addition to phosphorylation, IκBα is susceptible to other posttranslational modifications, such as SUMOylation (49). SUMOylation of IκBα makes it more stable and thus a better NF-κB inhibitor. Notably, the SUMOylation of IκBα is severely impaired if IκBα has previously been phosphorylated (28, 56). Consequently, phosphorylation of IκBα is associated with the activation of NF-κB responses, while IκBα SUMOylation correlates with inactivation of this pathway (49).

**FIG 6:**
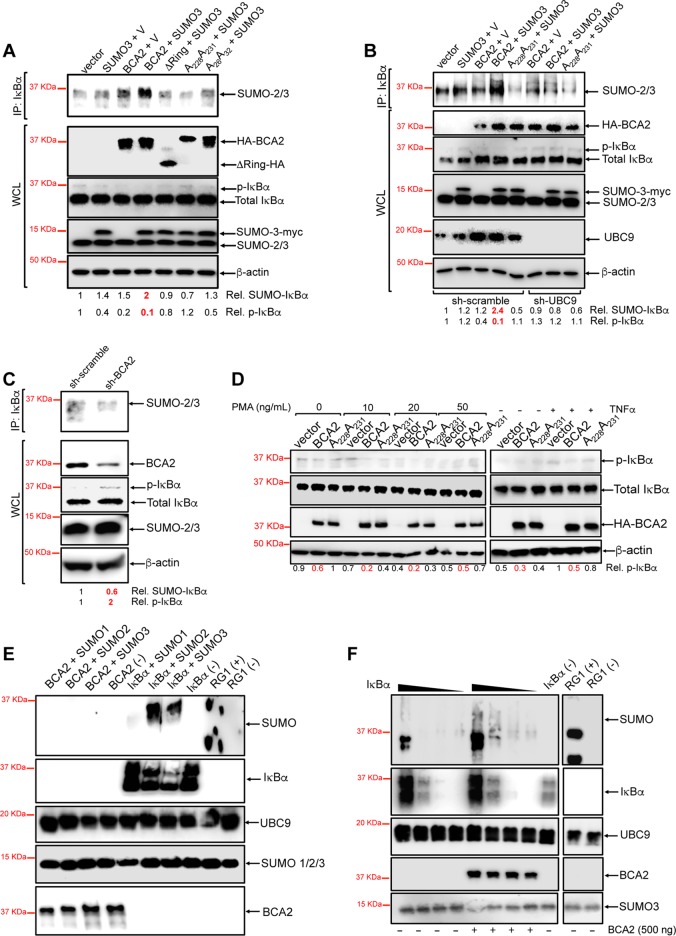
BCA2 inhibits NF-κB responses by enhancing the SUMOylation of IκBα. (A) 293T cells were cotransfected with a construct coding for HA-BCA2 or HA-BCA2 mutants and either an empty pcDNA5 vector (V) or a vector coding for SUMO-3-myc. Cell lysates were immunoprecipitated with an anti-IκBα antibody, and membranes were probed with an anti-SUMO-2/3 antibody. Relative amounts (Rel.) of SUMOylated IκBα and phosphorylated IκBα (p-IκBα) were quantified. (B) Similarly, the levels of SUMOylated and phosphorylated IκBα were evaluated in UBC9-depleted 293T cells. (C) The levels of IκBα SUMOylation were also examined in BCA2-depleted 293T cells. (D) The ability of BCA2 to prevent IκBα phosphorylation was also assessed in 293T cells expressing HA-BCA2, a BCA2 RING-defective mutant (A_228_A_231_), or an empty pcDNA5 vector in the presence and absence of PMA and TNF-α. The expression of all these constructs was verified by Western blotting. (E) *In vitro* SUMOylation studies were performed with purified BCA2 and IκBα in the presence of purified UBC9 and different SUMO isoforms. (F) Titration assays with decreasing amounts (3,000 to 3 ng) of IκBα in the presence and absence of BCA2. (+), positive control; (−), negative control (no Mg-ATP cofactors were added to the reaction mixture). WCL, whole-cell lysate. Representative data for three independent replicates are shown.

Since our data show that BCA2 needs a functional RING finger domain to inhibit NF-κB and that UBC9 is required to achieve this, we reasoned that BCA2 acts as an E3 SUMO ligase in the SUMOylation of IκBα. To test this hypothesis, the levels of SUMOylation and phosphorylation of IκBα were evaluated in the presence of BCA2. 293T cells were cotransfected with a construct coding for HA-BCA2 or HA-BCA2 mutants with impaired zinc finger and RING finger domains (A_26_ A_32_, A_228_ A_231_, and ΔRing) and either an empty vector or a vector coding for SUMO-3–myc. Forty-eight hours later, cells were harvested and endogenous IκBα was immunoprecipitated using a specific antibody. The pulled-down fraction was analyzed by Western blotting by probing membranes with an anti-SUMO-2/3 specific antibody. The whole-cell lysates were also analyzed by Western blotting for the expression of these constructs as well as β-actin, total IκBα, and phosphorylated IκBα, and the relative amounts of SUMOylated and phosphorylated IκBα were quantified. As expected, when SUMO-3–myc was exogenously expressed, there was an increase in the amounts of SUMO-IκBα compared to the vector control (Fig. 6A). Notably, the expression of HA-BCA2 alone or in combination with SUMO-3–myc resulted in a drastic increase in SUMO-IκBα (up to a 2-fold increase) and a significant reduction of phospho-IκBα (∼10-fold). By contrast, the degree of SUMOylation of IκBα was reduced to basal levels in the presence of BCA2 mutants with an impaired RING finger domain (Fig. 6A).

To further study the role of BCA2 in the SUMOylation of IκBα, additional assays were performed under conditions of UBC9 and BCA2 knockdown. Consistent with a role for UBC9 in IκBα SUMOylation (49), depletion of UBC9 in cells overexpressing HA-BCA2 led to a 3-fold decrease in SUMO-IκBα and an ∼10-fold increase in the levels of phosphorylated IκBα (Fig. 6B). In agreement with these results, the selective knockdown of BCA2 resulted in a 1.6-fold reduction in SUMO-IκBα and a 2-fold increase in phosphorylated IκBα (Fig. 6C). To verify that BCA2-mediated SUMOylation of IκBα prevents its phosphorylation, additional assays were performed under conditions of HA-BCA2 overexpression in the presence of phorbol myristate acetate (PMA), a protein kinase C (PKC) agonist that effectively activates the NF-κB pathway by enhancing IκBα phosphorylation (57–59). In addition, cells stimulated with TNF-α were included as a control. Under no stimulation, the basal levels of phosphorylated IκBα were lower in BCA2-overexpressing cells than in vector-treated cells or cells expressing the BCA2 RING-defective mutant (Fig. 6D, right), which is consistent with the data presented in Fig. 6A to C. Upon TNF-α stimulation, a 2-fold increase in phosphorylated IκBα was detected in cells transfected with an empty vector or expressing the catalytically inactive BCA2 mutant (Fig. 6D, right). Although overexpression of BCA2 did not completely block IκBα phosphorylation, the levels of phospho-IκBα remained markedly lower (∼2-fold) than in the other two instances (Fig. 6D, right), indicating that BCA2 effectively blocks the NF-κB pathway by impairing IκBα phosphorylation, although its effect is saturable upon strong stimulation. Similar results were obtained under conditions of PMA treatment. For this, cells expressing HA-BCA2, the BCA2 RING-defective mutant, and an empty vector were stimulated with dimethyl sulfoxide (DMSO) or increasing concentrations of PMA for 12 h, as previously reported (35, 60, 61). Of note, the amounts of phospho-IκBα declined over time across the assay, especially at higher concentrations of PMA (Fig. 6D, left). This may be due to the 12 h of incubation with the drug. PMA causes IκBα phosphorylation, and consequently, its ubiquitination and degradation (54, 55), so by the time of analysis, most of the phosphorylated form of IκBα may have been degraded. However, in the case of the assays with TNF-α, stimulation was performed for 2 h, so at this time point, the overall increase in phosphorylation is still evident. Despite this technicality, upregulation of BCA2 successfully reduced IκBα phosphorylation under conditions of PMA stimulation (∼2-fold), although BCA2's regulatory effect was saturated at the highest concentration of PMA (Fig. 6D, left).

To corroborate that BCA2 enhances the SUMOylation of IκBα and thereby works as an E3 SUMO ligase, *in vitro* SUMOylation assays were conducted. Purified IκBα and BCA2 were separately incubated with UBC9 and SUMO-1, SUMO-2, or SUMO-3 for 1 h at 37°C, and their SUMOylation levels were evaluated by Western blotting. RanGAP1 (RG1) was used as a positive control for SUMOylation. As negative controls, reactions omitting Mg-ATP cofactors were included. Consistent with previous reports (49), UBC9 was able to mediate IκBα SUMOylation in the absence of an E3 ligase. However, no evidence of BCA2 auto-SUMOylation was observed (Fig. 6E). To evaluate whether BCA2 enhances the efficiency of SUMOylation of IκBα, a titration assay was conducted with decreasing amounts of IκBα and constant levels of SUMO-3, BCA2, and UBC9. Remarkably, the rate of IκBα SUMOylation increased in the presence of BCA2 (Fig. 6F). Hence, these results reveal that both BCA2 and UBC9 participate in the SUMOylation of IκBα.

### BCA2 prevents the nuclear translocation of NF-κB.

**FIG 7:**
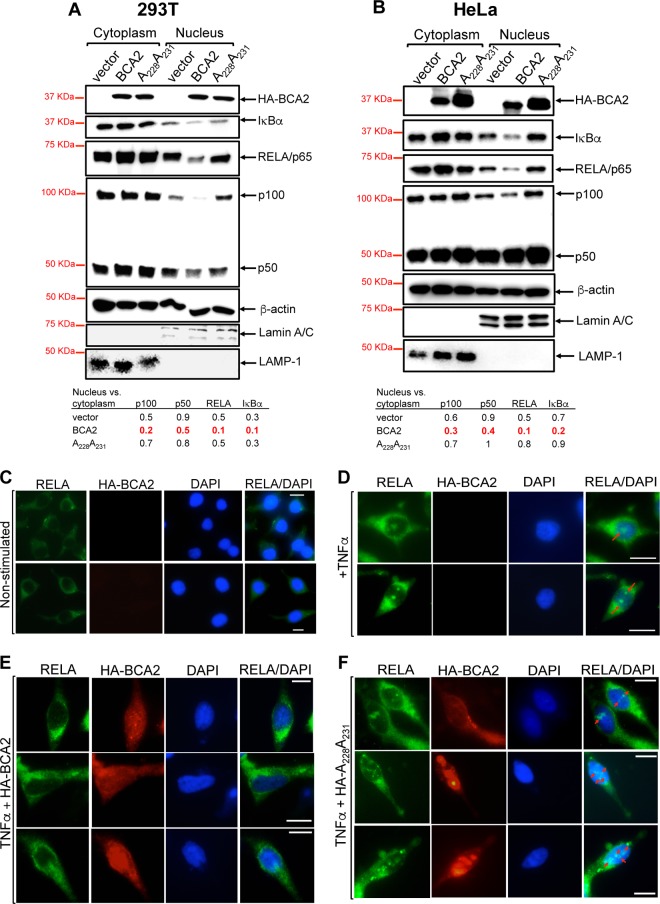
BCA2 prevents the nuclear translocation of NF-κB. 293T cells (A) and HeLa cells (B) were transfected with an empty vector (pcDNA5) or a vector coding for HA-BCA2 or an HA-BCA2 mutant with an impaired catalytic domain (A_228_A_231_). Cytoplasmic and nuclear fractions were separated and analyzed by Western blotting for the levels of β-actin, HA-BCA2, IκBα, RELA/p65, and p100/50 in each fraction. Lamin A/C was used as a nuclear marker and LAMP-1 as a cytoplasmic marker. The relative nuclear versus cytoplasmic levels of these proteins are provided below each blot. Cellular images were obtained from nonstimulated vector-transfected HeLa cells (C), TNF-α-stimulated vector-transfected HeLa cells (D), and TNF-α-stimulated HeLa cells expressing HA-BCA2 (E) or the RING-defective mutant (HA-A_228_A_231_) (F). Twenty minutes poststimulation, the cells were stained for HA-BCA2 (red), RELA (green), and nuclei (blue). The images are representative of four biological replicates. Bars, 10 μm. The arrows indicate nuclear RELA.

IκBα's ability to prevent the nuclear translocation of NF-κB is strengthened if it undergoes SUMOylation (49). The fact that BCA2 is involved in the SUMOylation of IκBα suggests that BCA2 ultimately inhibits the nuclear transport of NF-κB. To verify this, the nuclear versus cytoplasmic levels of NF-κB monomers and IκBα were examined. 293T and HeLa cells were transfected with an empty vector or a vector encoding HA-BCA2 or a BCA2 RING-defective mutant (A_228_ A_231_). Forty-eight hours later, cell lysates were differentially fractionated to obtain cytoplasmic and nuclear extracts. As previously reported, BCA2 and β-actin were both found in the cytoplasm and nucleus (40, 62). Consistent with a role for BCA2 in inhibiting the nuclear transport of NF-κB, the nuclear levels of RELA/p65, p100/p50, and IκBα were significantly reduced in cells expressing HA-BCA2 (ranging from 1.8- to-5-fold reduction) (Fig. 7A and B). By contrast, in cells expressing the BCA2 RING-defective mutant, the nuclear levels of NF-κB and IκBα were similar to those detected in vector-treated cells (Fig. 7A and B). These results were further corroborated by cellular images. For this, vector-transfected HeLa cells and cells expressing HA-BCA2 or the RING-defective mutant were stimulated with TNF-α for 20 min to induce NF-κB signaling. As previously reported, the majority of RELA was found in the cytoplasm in nonstimulated cells compared to vector-transfected and TNF-α-stimulated cells (Fig. 7C and D). Consistent with the data presented in Fig. 7A and B, a remarkable reduction of nuclear RELA was observed in cells expressing wild-type HA-BCA2 compared to cells expressing the BCA2 mutant (Fig. 7E and F). Therefore, these findings demonstrate that upregulation of BCA2 inhibits NF-κB by preventing its nuclear translocation.

## DISCUSSION

Innate antiviral factors are proteins that use unique mechanisms to prevent virus replication. Among the different anti-HIV factors identified so far, BCA2 exhibits dual anti-HIV activity. On one hand, BCA2 enhances BST2 restriction by targeting trapped virions for lysosomal degradation (20). On the other hand, BCA2 physically interacts with retroviral Gag proteins to promote their ubiquitination and lysosomal degradation in a BST2-independent manner (21). BCA2 is expressed endogenously in most tissues, but it is particularly upregulated in NK cells, CD8^+^ T cells, CD4^+^ T cells, dendritic cells, and monocytes, suggesting that its antiviral properties are of particular relevance for HIV-1 infection (available in NCBI Ace View [https://www.ncbi.nlm.nih.gov/ieb/research/acembly/av.cgi?db=human&term=RNF115] and the Human Protein Atlas databases [http://www.proteinatlas.org/search/rnf115]). BCA2 contains a zinc finger and a RING finger domain, and both are essential for its enzymatic activity (40, 42). Whereas the catalytic activity of BCA2 is dispensable for BST2-mediated restriction (20), an intact RING finger domain is required for BST2-independent antiviral activity (21), suggesting that the functional determinants for these two antiviral actions are genetically separable. Due to its dual function as a viral inhibitor, BCA2 acts as an important barrier for HIV-1 replication, and thus, it represents an attractive candidate for the development of drugs that would make HIV-1 more susceptible to BCA2 restriction. However, little is known about BCA2, its expression profile, and other potential immune-related activities or if HIV-1 has evolved mechanisms to overcome it.

Besides their roles as innate effectors in blocking virus replication, some antiviral factors can modulate, or are modulated by, the NF-κB signaling pathway, and thus, they are intimately connected to the innate sensing system (22–27). Recent studies reported that the promoter of *BCA2* contains responsive elements for NF-κB (28), suggesting that, similar to TRIM5α, BST2, and APOBEC3G, BCA2 is linked to the NF-κB pathway. The transcription factor NF-κB plays a major role in the regulation of immune and inflammatory responses (29, 30, 32), but it is also critical for HIV-1 transcription and replication (33, 34). In this study, we sought to investigate whether BCA2 is modulated by and/or modulates NF-κB and how its effects on this pathway can affect HIV-1 replication.

In agreement with previous reports (28, 41), our expression analyses show that BCA2 is upregulated in CD4^+^ T cells under conditions of NF-κB activation, such as IL-6 and TNF-α treatment (43, 44). To assess whether BCA2 has any regulatory effects on the NF-κB pathway, we performed luciferase-based reporter assays. Unlike BST2 or TRIM5α (22–26), no increase in NF-κB activity was detected under conditions of BCA2 upregulation. On the contrary, a dramatic reduction in NF-κB signaling was observed in the presence of factors that trigger NF-κB responses, indicating that BCA2 inhibits the NF-κB pathway, probably by serving as a sensor. Our mutagenesis studies revealed that BCA2 RING-defective mutants fail to block NF-κB activity, demonstrating that BCA2's E3 ligase activity is essential to hinder NF-κB signaling. To investigate if BCA2 could affect other inducible promoters, we assessed the ability of BCA2 to modulate interferon regulatory factors, such as IRF-1 and IRF-7. Unlike its effect on NF-κB, BCA2 induced IRF-1 signaling, whereas its upregulation led to a block of IRF-7-mediated responses, even in the presence of reported inducers. Since IRF-7 can be activated in response to NF-κB, it is likely that BCA2's role in the regulation of the NF-κB pathway consequently impacts IRF-7 signaling. Thus, beyond its function as a sensor for NF-κB, BCA2 plays additional roles in the modulation of innate signaling cascades.

According to the NF-κB canonical pathway, NF-κB is normally found in the cytoplasm associated with IκBα, an inhibitor that hides NF-κB's NLS (51–53). After cells are exposed to stimuli that trigger NF-κB signaling, a phosphorylation cascade is initiated, leading to the phosphorylation of IκBα, which in turn is targeted for ubiquitination and proteasomal degradation (54, 55). As a consequence of this, the NLS of NF-κB is uncovered, allowing translocation to the nucleus, where it binds to its target responsive genes (55). The cytoplasmic domain of HIV-1 gp41 induces the NF-κB canonical pathway by physically interacting with transforming growth factor beta (TGF-β) kinase 1 (TAK1) (38). In addition, HIV-1 also uses Nef to activate NF-κB (39). Thus, by virtue of triggering this innate pathway, HIV-1 ensures its transcription and replication (38). According to our luciferase assays, BCA2 outcompetes HIV-1 gp41 in the modulation of NF-κB, suggesting that BCA2 precisely blocks NF-κB components that are required to promote HIV-1 infectivity. In fact, the transcriptional capacity of HIV-1 was significantly reduced in 293T cells overexpressing BCA2 just 6 h after HIV-1 proviral transfection (63). Accordingly, the targeted depletion of BCA2 in both 293T and HeLa cells resulted in an ∼2.7-fold increase in the amount of HIV-1 RNAs compared to scrambled shRNA-treated cells. These observations are further supported by transcriptional and replication assays in Jurkat and primary cells, which demonstrate that BCA2 overexpression effectively delays proviral transcription and replication. Nonetheless, a decrease in BCA2 was observed for both vector-treated cells and cells overexpressing HA-BCA2 infected with HIV-1, suggesting that either the activation of additional innate signaling cascades in response to HIV-1 infection leads to a reduction in BCA2 or HIV-1 partially circumvents this barrier by decreasing the steady-state levels of the protein. Although we currently do not know if or how HIV-1 achieves this, the fact that the virus may have evolved to somewhat bypass the block imposed by BCA2 is in agreement with our previous work showing that HIV-1 Gag is less susceptible to BCA2 restriction than MLV or simian immunodeficiency virus (SIV) Gag protein (21). This might also explain why the effect of BCA2 on NF-κB activity measured by luciferase assays is more robust than its impact on proviral transcription.

Besides interacting with the E2 ubiquitin-conjugating enzyme UbcH5 (48), BCA2 was recently shown to bind UBC9 (28), the only E2 SUMO-conjugating enzyme currently known in humans. SUMOylation is a posttranslational modification that leads to the attachment of SUMO to target proteins. Similar to ubiquitination, SUMOylation requires sequential activation by E1, E2, and E3 enzymes, although UBC9 (E2) can mediate direct ligation to target molecules (64). Four different SUMO isoforms have been described in mammals (SUMO-1, -2, -3, and -4), and at least 100 different proteins have been reported to be targets for SUMOylation (65–68). Unlike ubiquitination, which may lead to a degradative pathway, SUMOylation can change the proteins' intracellular localization and interaction patterns with other protein and DNA molecules and may lead to other posttranslational modifications (69–71). Despite the importance of SUMOylation for cell homeostasis (69, 71, 72), the number of known E3 SUMO ligases is relatively low compared to the number of SUMOylated molecules, suggesting that there are still many unidentified E3 ligases (50). To examine if BCA2-mediated ubiquitination or SUMOylation was required to inhibit NF-κB, we assessed which of the two BCA2-interacting E2 enzymes was necessary to block this pathway. The targeted depletion of UbcH5 did not result in significant changes in the ability of BCA2 to limit NF-κB signaling nor, notably, did it affect HIV-1 transcription. However, the knockdown of UBC9 resulted in the inability of BCA2 to block HIV-1-induced NF-κB and, consequently, led to an increase in *nef*-containing transcripts. Therefore, BCA2 uses UBC9 to block NF-κB. These results were further corroborated by infectivity assays. Whereas the targeted depletion of UbcH5 led to a 2.7-fold increase in virus replication, the selective knockdown of UBC9 and BCA2 led to a 4- and a 5-fold increase in virus replication, respectively, by day 10 postinfection. Although UbcH5 is required for BCA2-mediated ubiquitination and subsequent lysosomal degradation of Gag, these results demonstrate that UBC9 is critical for HIV-1 restriction. Hence, unless UBC9 is required by any other anti-HIV-1 factor(s), our findings indicate that the BCA2-mediated inhibition of NF-κB accounts for most of BCA2's antiviral activity.

The fact that BCA2 uses UBC9 to successfully inhibit NF-κB suggests that BCA2 serves as an E3 SUMO ligase in the SUMOylation of IκBα, which would further impair the nuclear translocation of NF-κB (51–53). To test this hypothesis, we analyzed the SUMOylation levels of IκBα in the presence and absence of BCA2. Whereas BCA2 overexpression enhanced IκBα SUMOylation, which in turn resulted in a decrease in IκBα phosphorylation, depletion of BCA2 markedly reduced the levels of SUMOylated IκBα, increasing IκBα phosphorylation levels. These results were more evident under conditions of UBC9 knockdown, supporting the notions that (i) UBC9 appears to be the only E2-SUMO conjugating enzyme, (ii) cellular E3 ligases other than BCA2 may participate in the SUMOylation of IκBα, and (iii) UBC9 does not require E3 enzymes to ligate SUMO, although the reaction efficiency is lower than that of E3-mediated ligation (50). The ability of BCA2 to reduce IκBα phosphorylation is further supported by our PMA and TNF-α assays, in which the amounts of phosphorylated IκBα were markedly lower in BCA2-overexpressing cells than in cells expressing catalytically inactive BCA2 mutants. The role of BCA2 as an E3 SUMO ligase was confirmed in our *in vitro* SUMOylation studies, in which the rate of IκBα SUMOylation increased in the presence of BCA2. The SUMOylation of IκBα makes it a more potent inhibitor of the NF-κB pathway, since it further prevents the nuclear translocation of NF-κB. Consistent with this, a significant reduction in nuclear NF-κB components (RELA and p100/p50) was observed in cells overexpressing wild-type BCA2, but not in cells expressing RING-defective mutants. Similarly, a major reduction in nuclear IκBα was detected. When not bound to NF-κB, IκBα is constitutively transported to the nucleus to negatively regulate NF-κB-dependent transcription. This is achieved by preventing NF-κB–DNA interactions and promoting NF-κB transport back to the cytoplasm (73–75). Therefore, the reduced nuclear levels of IκBα in cells overexpressing BCA2 is in agreement with its role as a shuttle of NF-κB proteins to the cytoplasm, particularly if the NF-κB cascade needs to be inhibited. Thus, under conditions in which the NF-κB pathway is suppressed (i.e., BCA2 upregulation), the levels of nuclear IκBα and NF-κB components are reduced, and this corresponds to increased cytoplasmic levels of these proteins. These observations are further supported by cellular images, in which the nuclear localization of NF-κB is markedly lower in BCA2-overexpressing cells than in cells expressing BCA2 RING-defective mutants.

**FIG 8:**
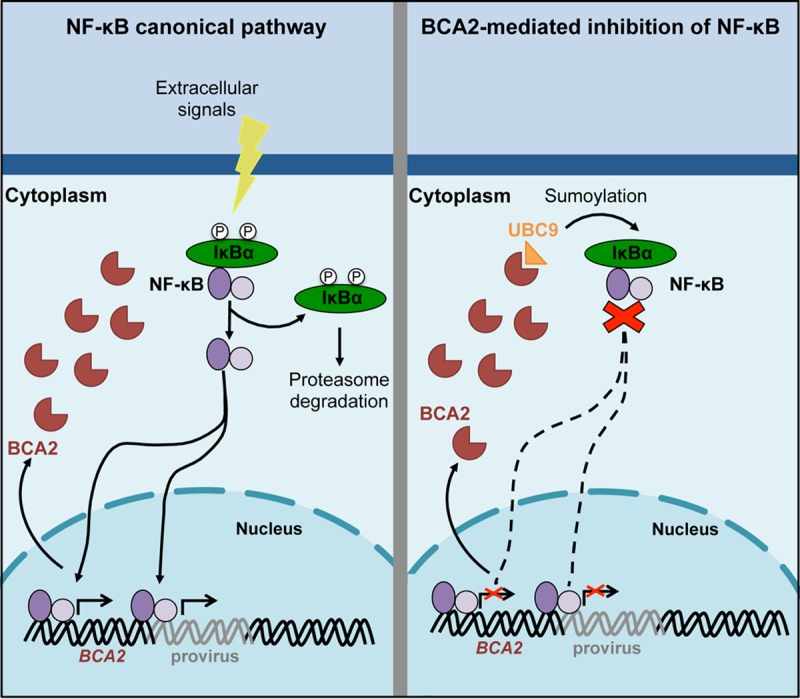
Proposed model of the role of BCA2 in the NF-κB pathway. (Left) *BCA2* is induced in response to NF-κB. Upon activation, NF-κB is translocated to the nucleus to regulate the expression of NF-κB-responsive genes, such as *BCA2*, and the HIV-1 provirus. (Right) Once BCA2 is synthesized, it primarily localizes in the cytoplasm, where its upregulation provides negative regulatory feedback on NF-κB signaling by enhancing the SUMOylation of IκBα, an inhibitor of NF-κB. This posttranslational modification makes IκBα a more potent inhibitor, further sequestering NF-κB elements in the cytoplasm, thereby contributing to HIV-1 proviral repression.

In summary, the findings presented here provide new insights into the anti-HIV activities of BCA2. We have demonstrated that *BCA2* is induced by NF-κB proinflammatory cytokines and that its upregulation leads to regulatory negative feedback on the NF-κB pathway. Specifically, BCA2 enhances the SUMOylation of IκBα, a previously unrecognized activity of BCA2. This modification leads to improved sequestration of NF-κB components in the cytoplasm, thereby preventing the expression of NF-κB-responsive genes. The BCA2-mediated inhibition of NF-κB outcompetes the ability of HIV-1 to trigger this signaling cascade, affecting HIV-1 gene expression and replication (Fig. 8). Nevertheless, our data also suggest that either the activation of additional immune pathways in response to viral infection or HIV-1 itself enhances BCA2 protein turnover or interferes with its expression. In conclusion, besides impairing HIV-1 assembly and release, BCA2 also represents an important barrier to HIV-1 replication at the transcriptional level.

## MATERIALS AND METHODS

### Plasmid DNA constructs. (i) BCA2 expression constructs.

Human BCA2 and ΔRing BCA2 were cloned into the pcDNA5 expression vector as previously described (21). Mutations at key residues in BCA2 were introduced by QuikChange site-directed mutagenesis (Stratagene, La Jolla, CA) according to the manufacturer's guidelines. The mutations introduced included amino acid substitutions in the BCA2 RING finger catalytic domain (C_228_ and C_231_ to A_228_ and A_231_) and the zinc finger domain (K_26_ and K_32_ to A_26_ and A_32_).

### (ii) BST2 expression constructs.

Human BST2 (hBST2) was cloned into pcDNA3 as previously described (76, 77).

### (iii) Moloney MLV (Mo-MLV) clone.

Vesicular stomatitis virus G glycoprotein (VSV-G)-pseudotyped MLVs were generated by transient transfection on 293T cells with the pNCS plasmid (Addgene, Cambridge, MA) and a construct coding for VSV-G (kindly provided by David T. Evans, University of Wisconsin).

### (iv) HIV-1 proviral clone.

Full-length HIV-1 NL4.3 proviral DNA (pNL4.3) was obtained through the NIH AIDS Reagent Program, Division of AIDS, NIAID, NIH, from Malcolm Martin.

### (v) CD8 constructs and luciferase vectors.

Constructs coding for CD8-STOP, CD8-gp41, the NF-κB-luciferase reporter plasmid, and the β-galactosidase expression vector were provided by Ronald C. Desrosiers, University of Miami, Miami, FL (38).

### (vi) SUMO-3–myc.

An expression vector (pcDNA3) coding for a human SUMO-3–myc-tagged construct was obtained through Addgene, Cambridge, MA.

### (vii) IRF-1 and IRF-7 constructs.

pIRF-1-Luc and pIRF-7-Luc vectors were obtained from Affymetrix, Santa Clara, CA.

### Transfections.

HEK293T and HeLa cells (American Type Culture Collection) were transfected using GenJet *in vitro* DNA transfection reagent (SignaGen Laboratories, Ijamsville, MD) following the manufacturer's instructions, including the suggested total DNA according to dish size, incubation time, and the ratio of GenJet DNA for optimal transfection efficiency. For all transfection experiments, cell viability was monitored to evaluate if the expression of the above-described constructs caused cellular toxicity or damage. No evidence of toxicity was observed. Viabilities were usually around 90%.

### Luciferase reporter assays.

For luciferase reporter assays, 3 × 10^5^ 293T cells were seeded on 6-well plates. Twenty-four hours later, the cells were transfected in triplicate with 500 ng of the expression vector of interest (BST2, HA-BCA2, HA-BCA2 mutants, HIV-1 NL4.3 proviral DNA, CD8-STOP, or CD8-gp41), 500 ng of luciferase reporter plasmid (NF-κB, IRF-1, or IRF-7), and 50 ng of the β-galactosidase plasmid (which was used to normalize the data for variations in transfection efficiency or cell numbers and/or for protein expression). In cases in which a combination of the plasmids of interest was required, the amount of total DNA was normalized by adding 500 ng of pcDNA5 empty vector to the simple transfection conditions. Forty-eight hours posttransfection, the cells were washed with ice-cold Dulbecco's phosphate-buffered saline (DPBS) and lysed in Reporter lysis buffer (Promega, Madison, WI) for 15 min at room temperature. The cell lysates were next centrifuged for 8 min at 16,000 × *g* at 4°C, and the supernatants were used to quantify luciferase activity (luciferase assay system; Promega, Madison, WI) and β-galactosidase activity (Galacto-Light Plus assay system; Applied Biosystems, Carlsbad, CA) in a plate reader, according to the respective manufacturer's instructions. Luciferase activity was normalized to β-galactosidase activity and expressed as NF-κB, IRF-1, or IRF-7 fold induction. Each luciferase reporter experiment was performed in three biological replicates and measured in technical replicates.

### shRNA knockdown of endogenous BCA2, UBC9, and UbcH5.

shRNA vectors to deplete BCA2 and a scrambled shRNA construct were provided in lentiviral vectors (pLKO.1) through the Thermo Scientific TRC Consortium (Broad Institute, Massachusetts Institute of Technology [MIT], and Harvard), as previously described (21). The following constructs were used for UBC9 depletion: TRCN0000368347, TRCN0000320448, and TRCN0000320374. The following constructs were used for UbcH5 knockdown: TRCN0000003381, TRCN0000003384, and TRCN0000003385. The targeted depletion of these genes was afforded by transient transfection on 293T and HeLa cells, according to the manufacturer's instructions (Thermo Scientific, Lafayette, CO). The knockdown of BCA2, UBC9, or UbcH5 was verified by Western blotting after the third round of transfection from 10^6^ cells and compared to the expression of β-actin. Cellular viability was also assessed. If viability was below 85%, cells were considered unsuitable for further analyses. Nevertheless, cellular viability was usually above 90%. Depleted cells were next used for luciferase assays or transcriptional assays. In this case, 1.2 × 10^6^ 293T or HeLa cells treated with sh-BCA2, sh-UBC9, sh-UbcH5, or sh-scramble RNA were transfected with 1,000 ng of full-length HIV-1 NL4.3 proviral DNA. HIV-1 transcriptional activity was assessed 6 and/or 18 h later by monitoring *nef* expression by RT-qPCR (see below).

For transcriptional and replication assays in Jurkat CD4^+^ T cells (American Type Culture Collection), VSV-G-pseudotyped lentiviral particles were produced to transduce each shRNA construct. The particles were generated by transient transfection on 293T cells using the psPAX2 packaging plasmid and pMD2.G envelope-expressing plasmid according to the manufacturer's instructions (Thermo Scientific, Lafayette, CO). Efficient knockdown of BCA2, UBC9, or UbcH5 was assessed by Western blotting after the third round of transduction from 10^6^ cells and compared to the expression of β-actin. Cellular viability was also assessed. If viability was below 85%, cells were considered unsuitable for further analyses. However, cellular viability was usually above 90%. Next, 10^6^ Jurkat CD4^+^ T cells were infected with 100 ng of p24 equivalents of HIV-1 NL4.3 and incubated for 3 h at 37°C. Infected cells were then washed and suspended in 5 ml of R10. HIV-1 transcriptional activity was monitored 18 h postinfection by measuring *nef*-carrying transcripts by RT-qPCR. In the case of replication assays, virus replication was monitored by p24 antigen capture enzyme-linked immunosorbent assay (ELISA) (Advanced Bioscience Laboratories, Rockville, MD) of virus particles released to the culture medium at the selected time points. Cell viability was also assessed at each time point.

Similar replication assays were performed in concanavalin A (ConA)-activated and IL-2-expanded human PBMCs (Zenbio Inc.) transduced with a retroviral empty vector (pQCXIP) or a retroviral vector encoding HA-BCA2. Forty-eight hours postransduction, 1 million (10^6^) cells were infected with 100 ng of p24 equivalents of HIV-1 NL4.3. Virus replication was monitored every 2 days by assessing the amounts of capsid p24 released to the culture supernatant by p24 antigen capture ELISA (Advanced Bioscience Laboratories, Rockville, MD). Cell viability was also assessed at each time point.

### Transcriptional analyses in Jurkat CD4^+^ T cells and human PBMCs.

One million Jurkat CD4^+^ T cells and human PBMCs from three different donors were transduced with a retroviral empty vector (pQCXIP) or pQCXIP-HA-BCA2. Prior to transduction, the PBMCs were concanavalin A (5 μg/ml) activated and IL-2 (10 U/ml) expanded for 3 days. Forty-eight hours after retroviral transduction, cellular viability was assessed. If viability was below 85%, cells were considered unsuitable for further analyses. However, cellular viability was usually above 90%. Next, 12 × 10^6^ cells were infected with 12 × 100 ng of p24 equivalents of HIV-1 NL4.3. After 3 h of infection, the cells were washed and seeded in 12-well plates at a concentration of 10^6^ cells/well. Samples were collected at 4, 6, 8, 10, 24, and 36 h postinfection. Cells were harvested, and total RNA was extracted, converted to cDNA, and analyzed for the presence of *nef*-containing RNAs by RT-qPCR as explained below. Additionally, the cell lysates derived from these experiments were analyzed by Western blotting for β-actin, endogenous BCA2, and p55/p24 to evaluate the presence/absence of Gag and capsid at the time of analysis.

### RT-qPCR assays. (i) RNA extraction and cDNA synthesis.

Total RNA was isolated and purified from transfected or infected cells (293T, HeLa, or Jurkat CD4^+^ T cells or PBMCs) using a Qiagen RNeasy minikit (West Sussex, United Kingdom) following the manufacturer's instructions. Total RNA purity and integrity were verified using a bioanalyzer (Genomics Unit, Center for Biotechnology, Texas Tech University) and with a Nanodrop spectrophotometer measuring optical density (OD). RIN values above 8 and OD at 260 nm/OD at 280 nm (OD_260_/OD_280_) absorption ratios between 1.8 and 2.0 were considered acceptable, and RNA samples were subsequently used for RT-qPCR analysis. cDNA was then synthesized from 1 μg of the total RNA using an iScript cDNA synthesis kit (Bio-Rad, Hercules, CA).

### (ii) RT-qPCR controls.

Prior to any study, each cDNA sample was tested for RNA quality (RNA quality assay RQ1 and RQ2), genomic DNA (gDNA) contamination, RT efficiency (RT control), and housekeeping gene (*GAPDH*) amplification by qPCR using the validated PrimePCR experimental control assay (Bio-Rad, Hercules, CA).

### (iii) *nef* qPCR.

For *nef* quantification of HIV-1-infected Jurkat CD4^+^ T cells and PBMCs, or HIV-1-transfected cells (293T or HeLa), the SYBR green-based real-time qPCR method was used. Each PCR was conducted in a 20-μl volume of 2× SsoAdvanced universal SYBR green supermix (Bio-Rad, Hercules, CA) with 20× PrimePCR *nef* primers (FW Nef primer, 5′-GTA CCA GTT GAG CCA GAT AAG G-3′, and RV Nef primer, 5′-GCT GTC AAA CCT CCA CTC TAA C-3′). The amplification conditions were set up as follows: 2 min at 95°C for initial activation and then 40 cycles at 95°C for 5 s and 60°C for 30 s, followed by melting analyses from 65 to 95°C (0.5°C increments). Melting curve analysis was performed, which for the *nef* primer set resulted in single-product-specific melting curves. No primer-dimer formations were observed during the 40 real-time PCR amplification cycles. PCR for each sample was performed in triplicate. In addition, the resulting real-time PCR product (10 μl) was loaded onto a 2% agarose gel and visualized by ethidium bromide staining (IBI Scientific, Peosta, IA). *nef* mRNA levels were normalized to *GAPDH* mRNA to generate a relative expression ratio. A fold change (up- or downregulation) of >2.0 was considered biologically relevant (63).

### SUMOylation and phosphorylation assays.

Six hundred thousand (6 × 10^5^) 293T cells were cotransfected with 2,000 ng of constructs expressing wild-type and mutant forms of HA-BCA2 (A_26_ A_32_, A_228_ A_231_, and ΔRing), along with an empty vector (pcDNA5) or a SUMO-3–myc construct. Forty-eight hours later, the cells were lysed with 300 μl of immunoprecipitation (IP) lysis buffer (Thermo Scientific, Rockford, IL) supplemented with a protease inhibitor cocktail (Sigma-Aldrich, St. Louis, MO, and Roche, Indianapolis, IN). In the case of PMA (10 to 50 ng/ml) and TNF-α (20 ng/ml) assays, cells were treated for 12 and 2 h, respectively, prior to the collection of whole-cell lysates. The cell lysates were then incubated on ice for 30 min. Next, samples were transferred to 1.5-ml tubes, and insoluble cell debris was removed by centrifugation at 16,000 × *g* at 4°C. An aliquot of each cell lysate (50 μl) was set aside to confirm expression of all of the above-mentioned constructs, as well as to assess IκBα phosphorylation by Western blot analysis (details on antibodies are provided in Table 1). The rest of the sample (250 μl) was used for immunoprecipitation assays. Samples for immunoprecipitation were incubated on a rotating platform for 1 h at 4°C with 25 μl of magnetic protein A-Sepharose beads (New England BioLabs, Ipswich, MA) to eliminate any nonspecific binding between the sample and the beads. Then, the samples were transferred to fresh 1.5-ml tubes and incubated on a rotating platform for an additional hour at 4°C with 1 μg of an anti-IκBα rabbit monoclonal antibody (Table 1). Magnetic protein A-Sepharose beads (25 μl) were then added, and the incubation was continued overnight at 4°C. Beads bound to the immunoprecipitated material were washed five times in IP lysis buffer (500 μl) and boiled in 2× SDS sample buffer (Sigma-Aldrich, St. Louis, MO). Proteins were separated by SDS-PAGE and detected by probing the membranes with an anti-SUMO-2/3 monoclonal antibody (Table 1). Membranes were imaged using a Li-Cor (Lincoln, NE) Fc Odyssey system, and band intensities were determined with Image Studio Li-Cor Odyssey Fc software.

**TABLE 1:** Antibodies, conditions, and sources of antibodies used in these studies

Protein	Primary antibody	Dilution	Source
β-Actin	Mouse monoclonal (AC-15) to β-actin	1:1,000	Abcam, Cambridge, MA
BCA2	Rabbit polyclonal to ZNF364	1:500	Abcam, Cambridge, MA
BST2	Mouse polyclonal to BST2	1:500	Abcam, Cambridge, MA
CD8	Mouse monoclonal (144B) to CD8	1:1,000	Abcam, Cambridge, MA
HA	Mouse monoclonal HA.11	1:1,000	Covance, Princeton, NJ
HIV-1 Gag p55/p24	Mouse monoclonal 183-H12-5C	1:1,000	AIDS Research and Reference Reagent Program, Division of AIDS, NIAID, NIH
IκBα	Rabbit monoclonal (E130) to IκBα	1:1,000	Abcam, Cambridge, MA
Lamin A/C	Mouse monoclonal (131C3) to Lamin A+C	1:1,000	Abcam, Cambridge, MA
LAMP-1	Rabbit polyclonal to LAMP-1	1:500	Abcam, Cambridge, MA
MLV p30	Rabbit polyclonal to MLV p30	1:500	Abcam, Cambridge, MA
p100/p50	Rabbit monoclonal (E381) to NF-κB p105/p50	1:1,000	Abcam, Cambridge, MA
Phospho-IκBα	Mouse monoclonal (39A1431) to IκBα (phospho S32 + S36)	1:1,000	Abcam, Cambridge, MA
RELA/p65	Rabbit monoclonal (E379) to NF-κB p65	1:1,000	Abcam, Cambridge, MA
SUMO-2/3	Mouse monoclonal (AT10F1) to Sumo-2 and -3	1:1,000	Abcam, Cambridge, MA
UBC9	Rabbit polyclonal to UBE2I/UBC9	1:500	Abcam, Cambridge, MA
UbcH5	Rabbit polyclonal to UBE2D1/UbcH5	1:500	Abcam, Cambridge, MA

### *In vitro* SUMOylation assays.

SUMOylation assays were performed with purified IκBα (3,000 to 3 ng; Abcam, Cambridge, MA) in the presence of purified UBC9 (200 nM; Abcam, Cambridge, MA) and/or BCA2 (500 ng; Abcam, Cambridge, MA) using a SUMOylation assay kit (Abcam, Cambridge, MA) for the generation of SUMOylated proteins *in vitro*. Negative-control reactions omitting Mg-ATP cofactors were included. RG1 was used as a positive control for *in vitro* SUMOylation reactions (78–80). The *in vitro* reaction was performed for 1 h at 37°C, following the manufacturer's instructions (Abcam, Cambridge, MA). Next, proteins were separated by SDS-PAGE and analyzed by Western blotting for the presence of SUMO conjugates.

### Western blots.

Cell lysates were prepared by harvesting in 2× SDS sample buffer (Sigma-Aldrich, St. Louis, MO). Samples were boiled for 5 min, separated by electrophoresis on SDS-8 to 12% polyacrylamide gels, and transferred to polyvinylidene difluoride (PVDF) membranes using a Trans-Blot SD transfer cell (Bio-Rad, Hercules, CA). The membranes were then blocked with 5% nonfat dry milk in PBS containing 0.05% Tween 20 for 1 h at room temperature and probed overnight at 4°C with one of the primary antibodies described in Table 1. After rinsing the PVDF membranes three times for 15 min each time in PBS-0.05% Tween 20, the membranes were probed with a horseradish peroxidase (HRP)-conjugated goat anti-mouse secondary antibody (Pierce, Rockford, IL) or an HRP-conjugated donkey anti-rabbit secondary antibody (Abcam, Cambridge, MA) at 1:2,000 dilution for 1 h. The blots were then rinsed three more times in PBS-0.05% Tween 20, treated with SuperSignal West Femto maximum-sensitivity substrate (Pierce, Rockford, IL), and imaged using a Li-Cor (Lincoln, NE) Odyssey Fc Imager 2800.

### Nuclear and cytoplasmic fractionations.

Five million (5 × 10^6^) 293T or HeLa cells were transfected with 5,000 ng of either an empty vector (pcDNA5) or a vector encoding HA-BCA2 (pcDNA5-HA-BCA2) or a BCA2 mutant (pcDNA5-HA-A_228_A_231_). Forty-eight hours later, the cells were lysed, and sequential extraction of proteins from specific compartments and/or organelles within the cell was performed using a ProteoExtract subcellular proteome extraction kit (S-PEK) (Millipore, Bedford, MA), following the manufacturer's instructions. The cytoplasmic fraction (F1) and nucleic protein fraction (F3) were analyzed by Western blotting using HA-, IκBα-, p100/p50-, and RELA/p65-specific antibodies (Table 1). Equal protein loading for the cytoplasmic and nuclear fractions was confirmed by blotting against β-actin (a cytoplasmic and nuclear marker). The purity of the nuclear and cytoplasmic fractions was determined by detecting lamin A/C (a nuclear marker) and LAMP-1 (a cytoplasmic marker), respectively.

### Fluorescence microscopy.

HeLa cells (2 × 10^4^ cells/well in an 8-well slide) were transfected with 100 ng of pcDNA5, pcDNA5-HA-BCA2, or pcDNA5-HA-A_228_A_231_. Forty-eight hours later, the cells were treated with 20 ng/ml of TNF-α for 20 min. Next, the cells were washed and fixed for 10 min in acetone-methanol and blocked for 20 to 60 min with 100 mM glycine diluted in 10% normal goat serum in PBS with 0.2% fish skin gelatin, 0.1% Triton X-100, and 0.02% sodium azide (10% NGS-PBS-FSG-Tx100-NaN_3_). The cells were then washed three times in 10% NGS-PBS-FSG-Tx100-NaN_3_ and stained for 1 h at room temperature. HA-BCA2 expression was tracked with a mouse monoclonal antibody to HA (IgG1) (Covance, Princeton, NJ) at a dilution of 1:250. To detect NF-κB components (RELA), we used a rabbit monoclonal anti-RELA antibody (Abcam, Cambridge, MA) at 1:200 dilution. The cells were subsequently stained with an Alexa-568-conjugated goat anti-mouse secondary antibody specific for IgG1 (1:1,000; Invitrogen, Grand Island, NY), with an Alexa-488-conjugated goat anti-rabbit antibody (1:1,000), and with DAPI (4′,6-diamidino-2-phenylindole) (1:5,000; Invitrogen, Grand Island, NY) to visualize cell nuclei. To ensure the specificity of the HA primary antibody and to assess that the Alexa-568 fluorophore was not nonspecifically stimulated by the other channels, vector-treated cells were also stained for HA-BCA2. After staining, the slides were washed and mounted with antiquenching mounting medium (Vector Laboratories, Inc.). Images were acquired using an Olympus BX41 fluorescence microscope.

### Statistical analysis.

All statistical calculations were performed with a two-tailed unpaired Student *t* test using Graph Pad Prism version 6.0 h. *P* values of ≤0.05 were considered statistically significant.
